# Aquibaculum arenosum gen. nov., sp. nov., a novel member of the family Rhodovibrionaceae, isolated from sea sand

**DOI:** 10.1099/ijsem.0.006458

**Published:** 2024-07-12

**Authors:** Soyeon Ahn, David Hyung-Sun Choi, Veeraya Weerawongwiwat, Jong-Hwa Kim, Ampaitip Sukhoom, Wonyong Kim

**Affiliations:** 1 Department of Microbiology, Chung-Ang University College of Medicine, Seoul 06974, Republic of Korea; 2 Faculty of Arts and Science, University of Toronto, Toronto, Ontario M5S 1A1, Canada; 3 Division of Biological Science, Faculty of Science, Prince of Songkla University, Songkhla 90112, Thailand

**Keywords:** *Alphaproteobacteria*, *Aquibaculum arenosum*, novel genus, novel species, *Rhodovibrionaceae*

## Abstract

A Gram-negative, non-motile, and creamy-white coloured bacterium, designated CAU 1616^T^, was isolated from sea sand collected at Ayajin Beach, Goseong-gun, Republic of Korea. The bacterium was found to grow optimally at 37 °C, pH 8.0–8.5, and with 1–5 % (w/v) NaCl. Phylogenetic analyses based on the 16S rRNA gene sequences placed strain CAU 1616^T^ within the order *Rhodospirillales*. The highest 16S rRNA gene sequence similarity was to *Fodinicurvata fenggangensis* YIM D812^T^ (94.1 %), *Fodinicurvata sediminis* YIM D82^T^ (93.7 %), *Fodinicurvata halophila* BA45AL^T^ (93.6 %) and *Algihabitans albus* HHTR 118^T^ (92.3 %). Comparing strain CAU 1616^T^ with closely related species (*Fodinicurvata fenggangensis* YIM D812^T^ and *Fodinicurvata sediminis* YIM D82^T^), the average nucleotide identity based on blast+ values were 69.7–69.8 %, the average amino acid identity values were 61.3–61.4 %, and the digital DNA–DNA hybridization values were 18.4–18.5 %. The assembled draft genome of strain CAU 1616^T^ had 29 contigs with an N50 value of 385.8 kbp, a total length of 3 490 371 bp, and a DNA G+C content of 65.1 mol%. The predominant cellular fatty acids were C_18 : 1_ 2−OH, C_19 : 0_ cyclo *ω*8*c*, and summed feature 8 (C_18 : 1_* ω*6*c* and/or C_18 : 1_* ω*7*c*). The major respiratory quinone was Q-10. Based on phenotypic, phylogenetic, and chemotaxonomic evidence, strain CAU 1616^T^ represents a novel genus in the family *Rhodovibrionaceae*, for which the name *Aquibaculum arenosum* gen. nov., sp. nov. is proposed. The type strain is CAU 1616^T^ (=KCTC 82428^T^=MCCC 1K06089^T^).

## Introduction

The family *Rhodovibrionaceae* is a member of the order *Rhodospirillales* [1], class *Alphaproteobacteria*, phylum *Pseudomonadota*. This family was first proposed by Hördt *et al*. [2]. *Rhodovibrio*, which was a genus in the family *Rhodospirillaceae*, has been classified in the *Rhodovibrionaceae*, along with *Limibacillus*, *Fodinicurvata*, *Limimonas*, *Pelagibius*, and *Tistlia*. At the time of writing, *Rhodovibrionaceae* comprises six validly published genera (https://lpsn.dsmz.de/family/rhodovibrionaceae) according to the List of Prokaryotic names with Standing in Nomenclature (LPSN). In general, members of the family *Rhodovibrionaceae* are characterized as Gram-negative and rod-shaped to spirillum. In this study, a novel member of the family *Rhodovibrionaceae* isolated from sea sand is described along with its phylogenetic and taxonomic position. Additionally, whole-genome analysis of strain CAU 1616^T^ has been explored, thereby providing deeper insight into ts metabolic products.

## ISOLATION AND ECOLOGY

Sea sand samples were collected at Ayajin Beach, Goseong-gun, Republic of Korea (38° 16′ 45.0″ N 128° 33′ 13.0″ E) in May 2020. It is famous for its abundance of rocks and algae, providing a conducive environment for the bacterial diversity of coastal ecosystems to thrive. We chose this site to carry out a microbial diversity project supported by the Korean Ministry of Environment. One gram of sea sand sample was serially diluted with NaCl 0.85 % (w/v) sterile water using a standard dilution plating technique for isolation. The samples were spread on Difco marine broth (MB) 2216 (Becton Dickinson) agar plates and incubated at 30 °C until visible individual colonies formed. The colonies with different morphologies were picked out and purified by repeated streaking on the same medium. CAU 1616^T^ was one of several isolated bacteria and used in taxonomic experiments.

The purified strain CAU 1616^T^ was preserved in MB supplemented with 25 % (v/v) glycerol at −80 °C. Strain CAU 1616^T^ was deposited in the Korean Collection for Type Cultures (KCTC) and the Marine Culture Collection of China (MCCC) with accession numbers KCTC 82848 and MCCC 1K06089, respectively. The most closely related type strains, namely *Fodinicurvata sediminis* YIM D82^T^ (=KCTC 22351^T^), *Algihabitans albus* HHTR 118^T^ (=KCTC 62395^T^), *Fodinicurvata fenggangensis* YIM D812^T^ (=DSM 21160^T^), and *Fodinicurvata halophila* BA45AL^T^ (=JCM 19075^T^), obtained from the KCTC, DSMZ (German Collection of Microorganisms, and Cell Cultures) and JCM (Japan Collection of Microorganisms), were used as reference strains and were grown following the recommendations of the culture collection.

## 16S rRNA GENE PHYLOGENY

Using a genomic DNA extraction kit (iNtRON Biotechnology), the genomic DNA of strain CAU 1616^T^ was isolated and purified. Using two bacterial universal primers (8F and 1525R [3]), the 16S rRNA gene was amplified and sequenced. The almost-complete 16S rRNA gene sequence was deposited in the GenBank database. The 16S rRNA gene sequence identity between strain CAU 1616^T^ (1446 bp) and its closely related strains selected by the EzBioCloud database (https://www.ezbiocloud.net/identify/) was examined. Phylogenetic trees were reconstructed with members of the family *Rhodovibrionaceae* and representative type species of the *Rhodospirillaceae*. mega7 software and Clustal W were used to align multiple sequences [4] and to perform phylogenetic tree reconstruction [5] using the neighbour-joining (NJ) method with the Jukes–Cantor model [6], the maximum-likelihood (ML) method [7], and the maximum-parsimony (MP) method [8]. The evolutionary distances were calculated based on the Jukes–Cantor model for the NJ tree and Kimura’s two-parameter model for the ML tree [9]. The topology of these phylogenetic trees was estimated based on 1000 random replications for each method [10].

**Fig. 1.**
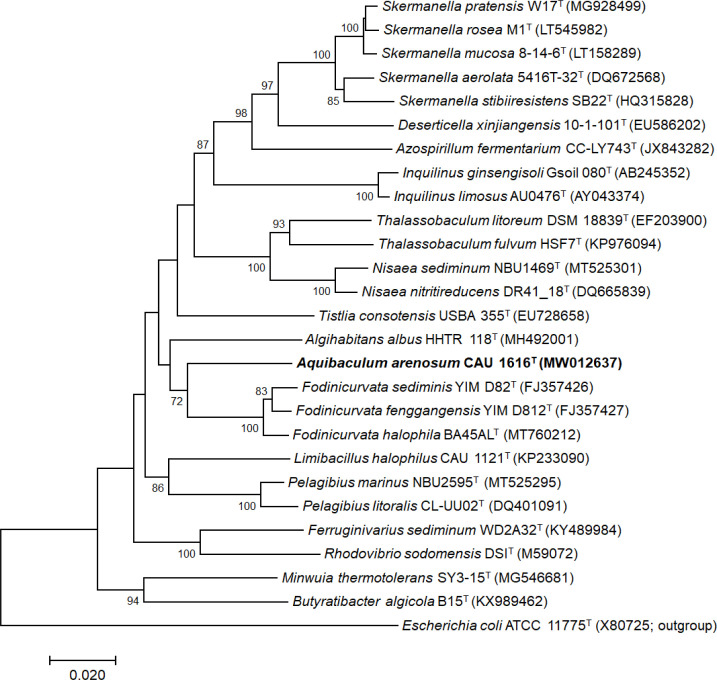
Neighbour-joining phylogenetic tree based on 16S rRNA gene sequences, showing the relationships between strain CAU 1616^T^ and closely related species. Branches conserved in maximum-likelihood and maximum-parsimony phylogenetic trees are marked with black circles (●) at the nodes. Bootstrap values (>70 %) are indicated as percentages of 1000 resampled datasets. *Escherichia coli* ATCC 11775^T^ (X80725) was used as an outgroup. The scale bar represents a 0.02 nucleotide sequence divergence.

Following a comparison of the 16S rRNA sequences in the EzBioCloud database, 16S rRNA sequences of the related strains were chosen based on their degree of similarity to reconstruct a phylogenetic tree. Strain CAU 1616^T^ is closely related to * F. fenggangensis* YIM D812^T^ (94.1 %), *F. sediminis* YIM D82^T^ (93.7 %), *F. halophila* BA45AL^T^ (93.6 %), and *A. albus* HHTR 118^T^ (92.3 %). The strains of the genus *Fodinicurvata* belong to the family *Rhodovibrionaceae*, and *A. albus* HHTR 118^T^ belongs to the family *Rhodospirillaceae*. The phylogenetic topologies were reconstructed with members of the family *Rhodovibrionaceae* and the type species of the family *Rhodospirillaceae*. *Escherichia coli* ATCC 11775^T^ (X80725) was selected as an outgroup. Based on 16S rRNA gene sequences, the phylogenetic tree showed that strain CAU 1616^T^ forms a phylogenetic lineage within the family *Rhodovibrionaceae* (Fig. 1). The trees reconstructed using the ML and MP algorithms supported this phylogenetic relationship (Figs S1 and S2, available in the online version of this article). The results of the phylogenetic analysis support that strain CAU 1616^T^ should be distinguished as a novel strain in the family *Rhodovibrionaceae*.

## GENOME, PHYLOGENETIC AND PHYLOGENOMIC ANALYSES

To determine the whole-genome sequences, DNA from CAU 1616^T^ was prepared using the Illumina TruSeq DNA PCR-Free kit. Sequencing was performed with an Illumina HiSeq sequencer and *de novo* assembly was performed using SPAdes version 3.15.0 at Macrogen (Seoul, Republic of Korea). Bacterial genomes were analysed via *k*-mer analysis using Jellyfish version 2.2.10 (http://www.genome.umd.edu/jellyfish.html), with genome size and genome heterozygosity used to estimate the *k*-mer frequency results through GenomeScope (http://qb.cshl.edu/genomescope/). The whole genome sequence was used to determined the DNA G+C content. Based on 92 core housekeeping genes from the whole genome, the up-to-date bacterial core gene (UBCG) was used to reconstruct the phylogenomic tree [11]. The expected functional genes were elucidated using Rapid Annotation using Subsystem Technology (rast; https://rast.nmpdr.org/) [12]. CAZyme genes were annotated by the dbCAN2 server (https://bcb.unl.edu/dbCAN2/blast.php) [13]. In addition, Clusters of Orthologous Groups (COGs) of proteins and their functional predictions were performed using eggNOG-mapper version 2 [14]. The genome’s secondary metabolism was verified using antiSMASH 7.0 [15]. CRISPRCasFinder was used to allow the identification of both CRISPR arrays and Cas proteins [16]. Strain CAU 1616^T^ and nine closely related type strains in the family *Rhodovibrionaceae* (except for *F. halophila*, for which the genomic data were absent) were used for the average nucleotide identity based on blast+ (ANIb), average amino acid identity (AAI), and digital DNA–DNA hybridization (dDDH) calculations. The ANIb and the AAI values were calculated with the JSspeciesWS web server (https://jspecies.ribohost.com/jspeciesws/#analyse) [17] and the AAI calculator (http://enve-omics.ce.gatech.edu/aai/), respectively. In addition, the dDDH values were computed using the Genome-to-Genome Distance Calculator (GGDC 3.0; https://ggdc.dsmz.de/ggdc.php) and the recommended formula 2 [18]. Comparative gene cluster analysis between strain CAU 1616^T^ and three type strains which had high 16S rRNA gene sequence similarity was performed using the OrthoVenn3 online server (https://orthovenn3.bioinfotoolkits.net/start/) [19].

**Fig. 2.**
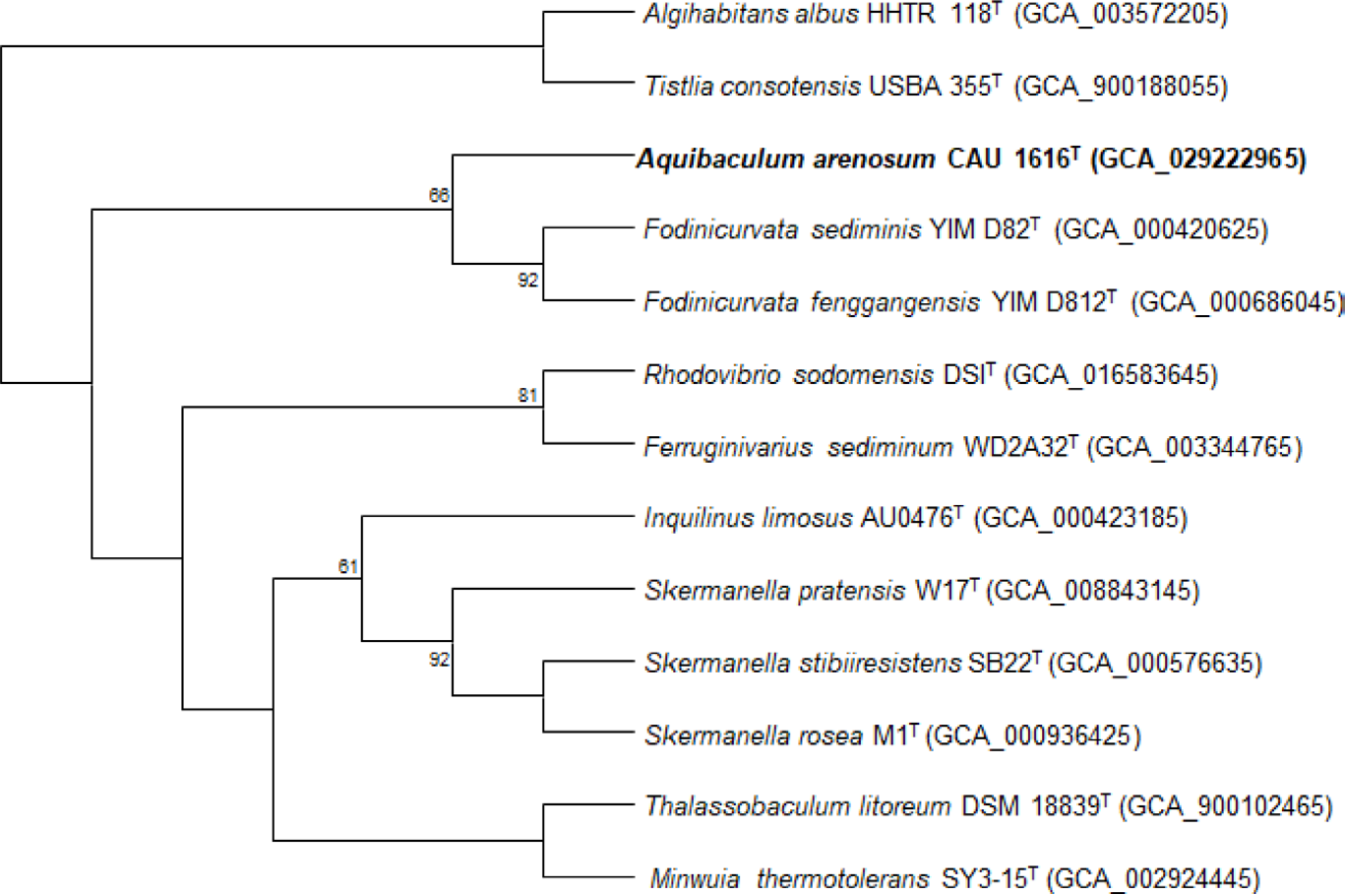
Phylogenetic tree reconstructed using core genomic data of strain CAU 1616^T^ and its closely related type strains. *Escherichia coli* ATCC 11775^T^ was used as an outgroup. Gene support index values of 92 UBCGs are given at branching points; only values >50 % are presented. Bar, 0.05 substitutions per nucleotide position.

The draft genome sequence of strain CAU 1616^T^ was 3.49 Mbp, with an N50 value of 385.8 kbp, and *k*-mer coverage of 360.8×. The draft genome sequence contained 29 contigs. The genomic DNA G+C content was 65.1 mol%. A total of 3272 genes were predicted, of which 46 genes were tRNA and four genes were rRNA (two 5S, one 16S, and one 23S rRNA). The differential genomic features between CAU 1616^T^ and the reference strains are presented in Table S1. The search of genomic features related to the genetic immunity of strain CAU 1616^T^ was performed using the CRISPRCasFinder server. One CRISPR array contains two spacers, and one CRISPR-Cas cluster was detected in strain CAU 1616^T^. Moreover, the phylogenetic tree reconstructed using the UBCG pipeline for the extraction of 92 core genes between the genome of strain CAU 1616^T^ and the members of *Rhodovibrionaceae* indicated that it is a novel strain from a novel genus in *Rhodovibrionaceae* (Fig. 2). The ANIb values between strain CAU 1616^T^ and its phylogenetically closest neighbours were 67.4–71.2 %. These values are lower than the threshold for species demarcation (95 %) [20]. The AAI values were 55.0–61.4 %, which are lower than the recommended threshold value of 65 % for bacterial genus delineation [21]. The dDDH values were 18.4–18.9 % with strains of the family *Rhodovibrionaceae*, namely *Fodinicurvata fenggangensis* YIM D812^T^, *Fodinicurvata sediminis* YIM D82^T^, *Limibacillus halophilus* CAU 1121^T^, *Limimonas halophila* IA16^T^, *Pelagibius litoralis* CL-UU02^T^, *Pelagibius marinus* NBU2595^T^, *Rhodovibrio salinarum* NCIMB 2243^T^, *Rhodovibrio sodomensis* DSI^T^, and *Tistlia consotensis* USBA 355^T^ (Table 1). The dDDH values under 18.9 % are much lower than the species threshold of 70 % recommended for species delineation [22]. The identified 3079 CDSs in the genome were classified into functional categories based on the COG database (Fig. S3). The most abundant COG categories were (E) amino acid transport and metabolism (425 genes), (C) energy production and conversion (226 genes), and (C) energy production and conversion (212 genes), which are associated with basic physiological functions and metabolism, except for (S) function unknown (555 genes). Comparison of COG annotation results between CAU 1616^T^ and closely related strains was provided in Table S2. The strain CAU 1616^T^ genome was assigned to functional categories based on the rast database. The composition of genes into subsystem categories is provided in Table S3. The predominant subsystems (over the 100 genes) of CAU 1616^T^ contained four groups, including amino acids and derivatives (279 genes; 20.65), protein metabolism (184 genes; 13.6 %), carbohydrates (164 genes; 12.1 %) and cofactors, and vitamins, prosthetic groups, and pigments (100 genes; 7.4 %). The genome annotation from rast showed the functional genes coding for potassium homeostasis. The coding genes were involved in bacterial survival, playing roles in osmoregulation, pH homeostasis, regulation of protein synthesis, enzyme activation, membrane potential adjustment, and electrical signalling, which supports that strain CAU 1616^T^ can survive in marine environments [23]. The potassium efflux system KefA protein/small-conductance mechanosensitive channel was identified in CAU 1616^T^ and all reference strains, but FKBP-type peptidyl-prolyl cis-trans isomerase SlyD (EC 5.2.1.8) and a large-conductance mechanosensitive channel was only identified in CAU 1616^T^ (Table S4). The compositions of the CAZymes in strain CAU 1616^T^ and the closely related strains was compared via the Carbohydrate-Active Enzymes Database (Table S5). The most abundant CAZyme of CAU 1616^T^ was glycosyltransferase (GT, 23), followed by glycoside hydrolases (GH, 16). The analogous CAZyme compositions of closely related strains were GT (16–35) and GH (8–14). Notably, these high numbers of the predicted CAZymes correlated with the number of COG functional categories (G, carbohydrate transport and metabolism]. The secondary metabolite clusters were obtained in the antiSMASH. The strain CAU 1616^T^ genome consists of six putative BGCs (RRE-containing, T3PKS, NAGGN, NRPS-like, terpene, T1PKS) revealed by antiSMASH analysis. The core and additional biosynthetic genes were predicted for secondary metabolite biosynthetic gene clusters (smBGCs) (Table S6). Moreover, each identified gene cluster from antiSMASH analysis was compared against the NCBI database using protein-protein blast (blastp). The transcription factor binding sites (TFBS) were predicted in CAU 1616^T^, such as zinc-responsive repressor (*ZuR*), represses ribonucleotide reductase encoding genes (*NrdR*), antibiotic production activator (*AbrC3*), repressor of DNA damage response (*LexA*), and regulator of arginine biosynthesis genes (*ArgR*). In addition, the CAU 1616^T^ genome presented genes potentially responsible for resistance to aminoglycoside antibiotic (*AAC(3)-IIb*) in its chromosome. The annotation of orthologous gene clusters of CAU 1616^T^ and phylogenetically closely related species was compared via a Venn diagram (Fig. S4). Strain CAU 1616^T^ shared 1632 orthologous gene clusters with three reference strains. Strain CAU 1616^T^ has 28 orthologous gene clusters, which did not overlap with all reference strains.

**Table 1.** The ANIb, AAI and dDDH values between CAU 1616^T^ and the strains of the family *Rhodovibrionaceae* (except *Fodinicurvata halophila* BA45AL^T^, for which genomic data was missing in the NCBI database)

Type species	ANIb (%)	AAI (%)	dDDH (%)
*Fodinicurvata fenggangensis* YIM D812^T^	69.8	61.3	18.5
*Fodinicurvata sediminis* YIM D82^T^	69.7	61.4	18.4
*Limibacillus halophilus* CAU 1121^T^	67.4	57.6	18.9
*Limimonas halophila* IA16^T^	68.6	55.0	18.0
*Pelagibius litoralis* CL-UU02^T^	69.2	56.9	18.3
*Pelagibius marinus* NBU2595^T^	70.3	57.6	18.3
*Rhodovibrio salinarum* NCIMB 2243^T^	68.7	55.9	18.9
*Rhodovibrio sodomensis* DSI^T^	69.2	55.4	18.1
*Tistlia consotensis* USBA 355^T^	71.2	59.9	19.0

## PHYSIOLOGY AND CHEMOTAXONOMY

Growth of strain CAU 1616^T^ was examined on different media, including marine agar (MA), tryptic soy agar (TSA), brain heart infusion (BHI) agar, nutrient agar (NA), Luria–Bertani (LB) agar, and glucose–yeast extract–agar (GYEA) (all Difco) at 30 °C for 4 days. The temperature range for growth was tested at 4, 10, 20, 30, 37, 40, 42 and 45 °C in MB. The effect of NaCl tolerance on growth was examined in nutrient broth (NB) with various NaCl concentrations (0 %–15 %; 1 % intervals, w/v) [24]. The range of pH for growth was investigated from pH 4.5 to 12.0(0.5 pH unit intervals) using sterile solution buffer (10 mM sodium acetate–acetic acid; pH 4.5–5.5, 10 mM Tris–HCl; pH 6.0–9.0, and 10 mM NaHCO3/Na2CO3; pH 9.5–12.0). Gram stain was examined using a kit (bioMérieux), and gliding motility was examined by the hanging-drop method. The cellular morphology of CAU 1616^T^ was observed by light microscopy (model DM 1000, Leica) and transmission electron microscopy (TEM; model JEM 1010, jeol). Catalase activity was determined by adding 3 % H_2_O_2_ solution (v/v), and oxidase activity was assessed using 1 % tetramethyl*-p*-phenylenediamine (w/v). Hydrolysis of starch and casein was determined following the method of Smibert and Krieg [25]. Biochemical features and enzyme activities were tested using API 20E, API 20NE, API ZYM, and API 50CH strips (all bioMérieux), according to the manufacturer’s instructions. The respiratory quinones were purified by TLC and analysed by HPLC as described by Da Costa *et al*. [26]. For the cellular fatty acid analysis, bacterial biomass of strain CAU 1616^T^ and the reference strains was harvested after culture on MA for 3 days at 30 °C. Fatty acid methyl esters were analysed via the technique described by Pineiro-Vidal *et al*. [27]. In addition, the peaks were analysed using the Microbial Identification software package (moore library version 5.0; midi database TSBA6).

Strain CAU 1616^T^ was a Gram-stain-negative, non-spore-forming, motile bacterium and showed coccus-shaped morphology (Fig. S5). After 3 days at 30 °C under aerobic conditions, colonies were creamy white in colour, circular form, convex, smooth, and opaque. Cells grew aerobically on MA, BHI, and LB agar, but not on GYEA, NA, and TSA medium. Growth occurred at 20–42 °C (optimum, 37 °C), pH 8.0–8.5, and in the presence of 1–10 % (w/v) NaCl (optimum, 1–5 %). Strain CAU 1616^T^ was positive for catalase and oxidase and negative for hydrolysis of casein and starch. The API ZYM assay results were positive for esterase (C4), esterase lipase (C8), leucine arylamidase, valine arylamidase, trypsin, and naphthol-AS-BI-phosphohydrolase. Using API 50CH tests, the result for potassium 5-ketogluconate was positive. The API 20E/20NE assay results were positive for β-glucosidase. Detailed morphological, physiological, biochemical, and cultural characteristics of CAU 1616^T^ and representative genera of the family *Rhodovibrionaceae* are shown in Table 2. The predominant respiratory quinone of strain CAU 1616^T^ was Q-10, the same as that in *Fodinicurvata sediminis* YIM D82^T^, *Pelagibius litoralis* CL-UU02^T^, *Limibacillus halophilus* CAU 1121^T^, and *Limimonas halophila* IA16^T^. This differed from *Tistlia consotensis* USBA 355^T^, which contains both Q-10 and Q-9, and from *Rhodovibrio salinarum* ATCC 35394^T^, which contained both Q-10 and MK-10. The major fatty acid types of CAU 1616^T^ (>10 %) were C_18 : 1_ 2−OH (15.1 %), C_19 : 0_ cyclo *ω*8*c* (19.9 %), and summed feature 8 (C_18 : 1_* ω*6*c* and/or C_18 : 1_* ω*7*c*; 60.1 %). These results were similar to those of its closely phylogenetic neighbours, as shown in Table 3.

**Table 2.** Characteristics that differentiate strain CAU 1616^T^ from representative genera of the family *Rhodovibrionaceae* Strains: 1, CAU 1616^T^; 2, *Fodinicurvata sediminis* YIM D82^T^ [28]; 3, *Tistlia consotensis* USBA 355^T^ [29]; 4, *Pelagibius litoralis* CL-UU02^T^ [30]; 5, *Limibacillus halophilus* CAU 1121^T^ [31]; 6, *Limimonas halophila* IA16^T^ [32]; 7, *Rhodovibrio salinarum* ATCC 35394^T^ [33]. All strains are Gram-negative. Data for the type materials were from the references. +, Positive; –, negative; na, not available.

Characteristics	1	2	3	4	5	6	7
Colony colour	Cream-white	Cream-white	Beige	Cream	Cream	Non-pigmented	Red
Cell shape	Coccus	Rod and vibrioid	Slightly curved rod	Slightly curved rod	Short rod	Rod	Rod and vibrioid
Cell size (μm)	0.9–1.0×1.0–1.1	0.3–0.5×0.7–1.5	0.6–0.7×3.0–3.5	0.5–1.0×1.2–2.5	0.3–0.45×1.0–2.0	0.1–0.2×1.5–2.0	1.0–3.0×0.3–0.5
Motility	+	–	+	+	–	–	+
Flagella	–	–	–	+	–	–	+
Temperature range for growth (°C)	20–42	15–42	20–40	15–33	20–40	30–50	20–45
Optimum temperature for growth (°C)	37	28	30	28–30	37	40	42
pH range for growth	8.0–8.5	6.5–8.5	5.0–8.0	6.0–11.0	6.5–10.5	6.0–8.0	na
Optimum pH for growth	8.0–8.5	7.5	6.5–6.7	7.0–8.0	6.5	7.0	7.5–8.0
NaCl range for growth (%, w/v)	1–10	1.5–20	0–4	2–6	0–5	15–30	2–24
Optimum NaCl for growth (%, w/v)	1–5	5	0.5	3–4	2	20	6–18
Nitrate reduction	–	+	+	+	+	–	na
Catalase activity	+	+	–	+	+	+	na
Oxidase activity	–	–	na	+	+	+	na
Major quinones	Q-10	Q-10	Q-10, Q-9	Q-10	Q-10	Q-10	Q-10, MK-10
Major fatty acids	C_18 : 1_ 2−OH, C_19 : 0_ cyclo *ω*8*c*, summed feature 8 (C_18 : 1_* ω*6*c* and/or C_18 : 1_* ω*7*c*)	C_19 : 0_ cyclo *ω*8*c*, summed feature 8 (C_18 : 1_* ω*6*c* and/or C_18 : 1_* ω*7c)	C_19 : 0_ cyclo*ω*8*c*, C_18 : 1_* ω*7*c*, C_18 : 0_	C_18 : 1_* ω*7*c*, C_18 : 0_ 3-OH, C_19 : 0_ cyclo *ω*8*c*	C_18 : 1_* ω*7*c*, C_19 : 0_ cyclo *ω*8*c*	C_19 : 0_ cyclo *ω*7*c*, C_18 : 0_, C_18 : 1_*ω*7*c*, C_16 : 0_	na

**Table 3.** Fatty acid composition (% of total) of strain CAU 1616^T^ and its closely phylogenetic neighbours Strains: 1, CAU 1616^T^; 2, *Fodinicurvata sediminis* YIM D82^T^; 3, *Fodinicurvata fenggangensis* YIM D812^T^; 4, *Fodinicurvata halophila* BA45AL^T^; 5, *Algihabitans albus* HHTR 118^T^. −, Not detected; tr, trace (<1 %). Only fatty acids making up >1 % of the total are shown; major fatty acids (>10 %) are shown in bold.

Fatty acids	1	2	3	4	5
**Saturated**					
C_10 : 0_	−	tr	tr	tr	–
C_12 : 0_	–	–	–	–	4.4
C_16 : 0_	tr	5.2	6.1	5.0	2.0
C_17 : 0_	−	−	tr	tr	–
C_18 : 0_	tr	3.7	2.9	3.5	7.6
**Unsaturated**					
C_17 : 1_* ω*7*c*	1.7	tr	tr	tr	1.7
C_18 : 1_ ω7*c* 11-methyl	−	1.2	7.2	1.2	1.8
C_17 : 0_ cyclo	−	−	1.3	−	–
C_19 : 0_ cyclo *ω*8*c*	**19.9**	**41.8**	**49.9**	**44.3**	tr
C_20 : 2_* ω*6,9*c*	tr	tr	tr	tr	tr
C_20 : 1_* ω*7*c*	−	−	−	−	1.2
**Hydroxy**					
C_16 : 0_ 2-OH	−	−	tr	tr	2.5
C_18 : 0_ 2-OH	−	tr	tr	tr	–
C_18 : 0_ 3-OH	tr	1.4	1.1	1.5	1.2
C_18 : 1_ 2-OH	**15.1**	9.1	5.7	7.1	**14.4**
Summed features*					
2	–	–	–	–	1.3
3	−	−	tr	−	–
8	**60.1**	**35.6**	**22.7**	**34.9**	**61.6**

*Summed Ffeatures are fatty acids that cannot be resolved reliably from another fatty acid using the chromatographic conditions chosen. The MIDImidi system groups these fatty acids together as one feature with a single percentage of the total. Summed feature 22 was listed as of C_16 : 1_ Iiso I and/or C_14 : 0_ 3-OH. Summed feature 33 was listed as of C_16 : 1_* ω*6*c* and/or C_16 : 1_* ω*7*c*. Summed feature 88 was listed as of C_18 : 1_* ω*6*c* and/or C_18 : 1_* ω*7*c*.

## TAXONOMIC CONCLUSION

The phenotypic and chemotaxonomic analysis revealed that strain CAU 1616T shares some common characteristics with members of the family *Rhodovibrionaceae*, but also exhibits significant differences from other genera within this family. These findings suggest that CAU 1616^T^ represents a novel genus of *Rhodovibrionaceae*. Based on morphological, physiological, phylogenetic, phylogenomic, biochemical, genomic, and chemotaxonomic differences between strain CAU 1616^T^ and the type strains of other phylogenetically closely related genera of the family *Rhodovibrionaceae*, we have determined that strain CAU 1616^T^ represents a novel species in a novel genus, which we have named *Aquibaculum arenosum* gen. nov., sp. nov.

## DESCRIPTION OF *AQUIBACULUM* GEN. NOV.

*Aquibaculum* (A.qui.ba’cu.lum. L. fem. n. *aqua*, water; L. neut. n. *baculum*, stick; N.L. neut. n. *Aquibaculum*, a rod-shaped bacterium, isolated from sea water).

Cells are Gram-stain-negative, aerobic, non-spore-forming, motile, and coccus-shaped. Cells are positive for catalase and oxidase. Growth occurs at 20–37 °C (optimal at 37 °C), 1–10 % NaCl (optimal at 1–5 %), and pH 8.0–8.5. The genomic DNA G+C content is 65.1 mol%. The predominant respiratory quinone is Q-10, and the major fatty acids include C_18 : 1_ 2−OH, C_19 : 0_ cyclo *ω*8*c*, and summed feature 8 (C_18 : 1_* ω*6*c* and/or C_18 : 1_* ω*7*c*). Phylogenetically, the genus is affiliated to the family *Rhodovibrionaceae* of the order *Rhodospirillales*. The type species is *Aquibaculum arenosum*.

## Description of *AQUIBACULUM ARENOSUM* SP. NOV.

*Aquibaculum arenosum* (a.re.no’sum. L. neut. adj. *arenosum*, sandy, dwelling in sand).

Cells are Gram-stain-negative, aerobic, non-spore-forming, motile, and coccus-shaped (0.9–1.0 µm long and 1.0–1.1 µm wide). The creamy white colour colonies are circular, convex, smooth, and opaque on MA medium after 3 days of incubation at 30 °C in aerobic conditions. Optimal growth occurs at 37 °C, pH 8.0–8.5, and 1–5 % (w/v) NaCl. The results of casein and starch hydrolysis are negative. Cells are positive for catalase and oxidase. The predominant respiratory quinone is Q-10, and the major fatty acids include C_18 : 1_ 2-OH, C_19 : 0_ cyclo *ω*8*c*, and summed feature 8 (C_18 : 1_* ω*6*c* and/or C_18 : 1_* ω*7c). The type strain, CAU 1616^T^ (=KCTC 82428^T^=MCCC 1K06089^T^), was isolated from a sea sand sample collected near Ayajin Beach, Goseong-gun, Republic of Korea. The G+C content of the genomic DNA is 65.1 mol%. The 16S rRNA gene and genomic sequences of strain CAU 1616^T^ have been deposited under the GenBank/EMBL/DDBJ accession numbers MW012637 and JARHUD000000000, respectively.

## supplementary material

10.1099/ijsem.0.006458 Uncited Supplementary Material 1.

## Abbreviations

AAI: average amino acid identity
ANIb: average nucleotide identity based on blast+
BHI: brain heart infusion
COG: cluster of orthologous groups
dDDH: digital DNA–DNA hybridization
GYEA: glucose–yeast extract–agar
LB: Luria–Bertani
MA: marine agar
NA: nutrient agar
RAST: Rapid Annotation using Subsystem Technology
TSA: tryptic soy agar
UBCG: up-to-date bacterial core genome
