# Supplementary material for: Aquibaculum arenosum gen. nov., sp. nov., a novel member of the family Rhodovibrionaceae, isolated from sea sand
Source: Int J Syst Evol Microbiol. 2024 Jul 12;74(7):006458. doi: 10.1099/ijsem.0.006458 (PMC11316597; doi:10.1099/ijsem.0.006458)
Supplement: Uncited Supplementary Material 1. [file ijsem-74-06458-s001.pdf]

Description of *Aquibaculum arenosum* gen. nov., sp. nov., a new member in the family *Rhodovibrionaceae*, isolated from sea sand

Soyeon Ahn[1] • David Hyung-Sun Choi[2] • Veeraya Weerawongwiwat[1] • Jong-Hwa Kim[1] • Ampaitip Sukhoom[3] • Wonyong Kim[1*]

[1]Department of Microbiology, Chung-Ang University College of Medicine, Seoul 06974, Republic of Korea

[2]Faculty of Arts and Science, University of Toronto, 27 King's College Cir, Toronto, Ontario M5S 1A1, Canada

[3]Division of Biological Science, Faculty of Science, Prince of Songkla University, Songkhla 90112, Thailand

[*]**Correspondence**:

Professor Wonyong Kim, Ph.D.

Department of Microbiology, Chung-Ang University College of Medicine, Seoul 06974, Republic of Korea. Tel: +82-2-820-5685; E-mail: kimwy@cau.ac.kr, ORCID ID: 0000-0001-9649-3919.

Fig S1. Maximum-likelihood (ML) phylogenetic tree based on 16S rRNA gene sequences showing the similarity between strain CAU 1616^T and closely related taxa. Bootstrap values (>70%) are indicated as percentages of 1,000 replicated datasets. *Escherichia coli* ATCC 11775^T (X80725) was used as an outgroup. The scale bar represents a 0.02 nucleotide sequence divergence.

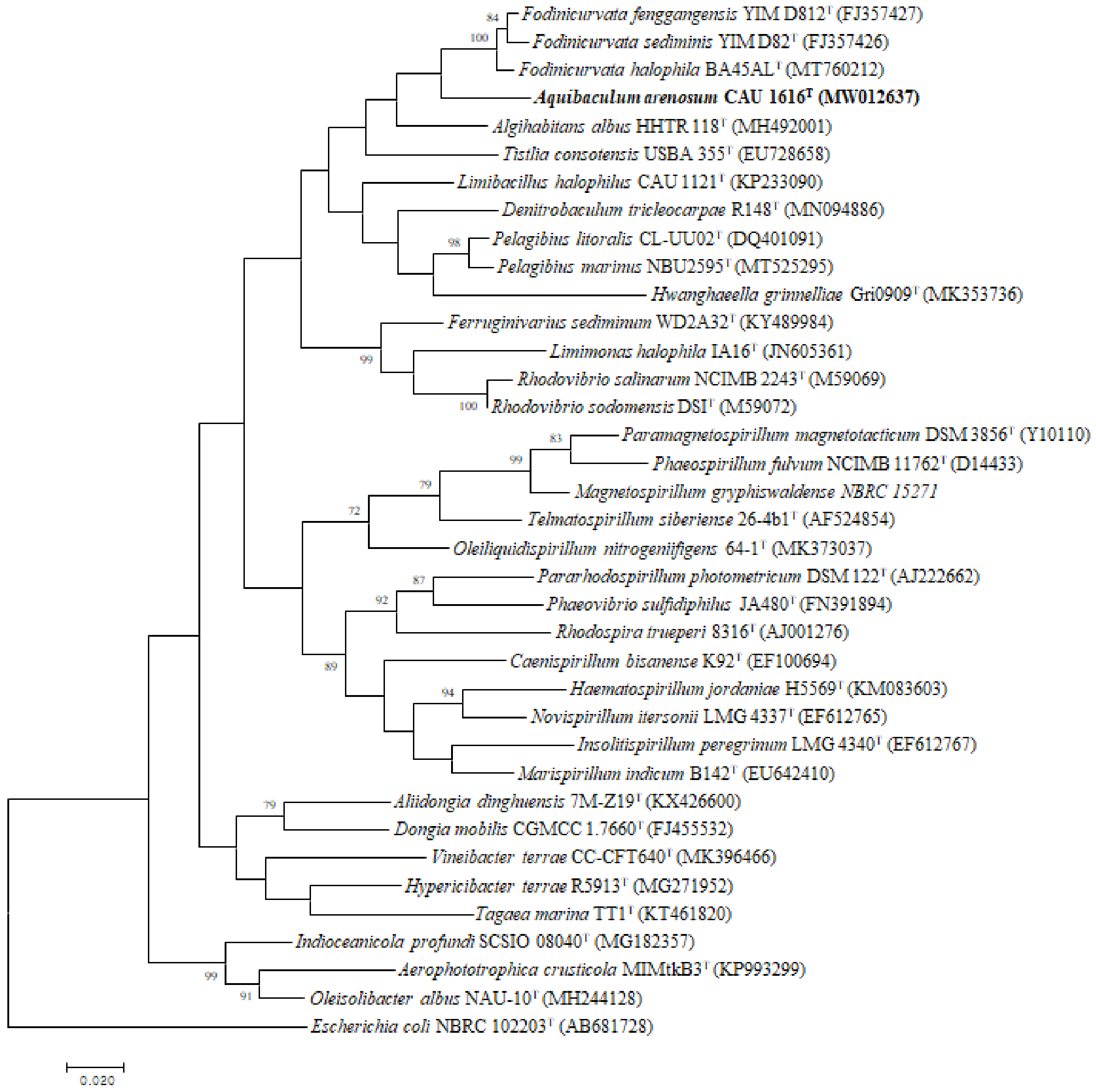

Fig S2. Maximum-parsimony (MP) tree based on the 16S rRNA gene sequence of CAU 1616^T. Bootstrap values are shown in percentages of 1,000 replicates when greater than 70%. *Escherichia coli* ATCC 11775^T (X80725) was used as an outgroup.

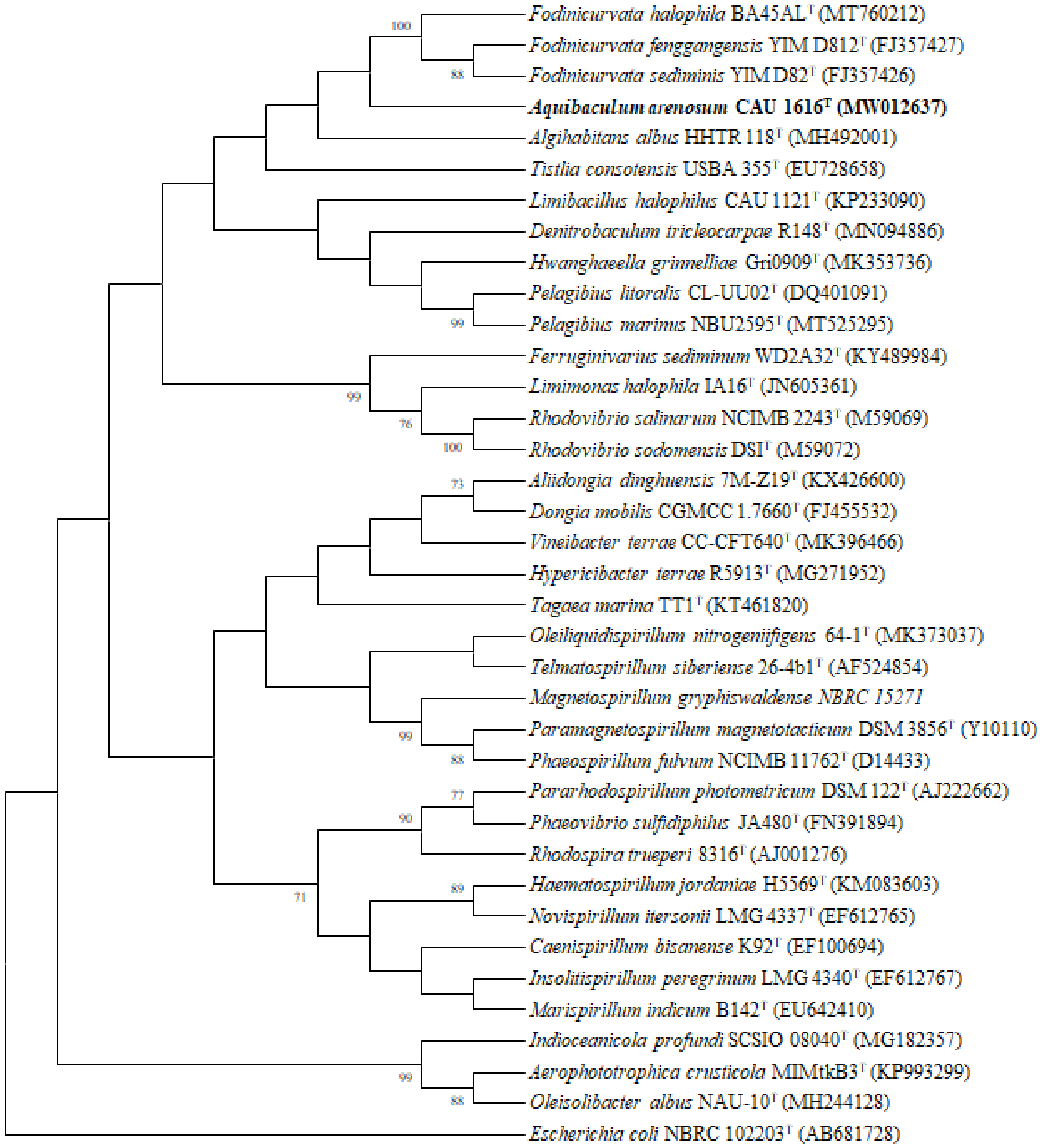

Fig S3. Distribution of COG categories in the genome of strain CAU 1616^T.

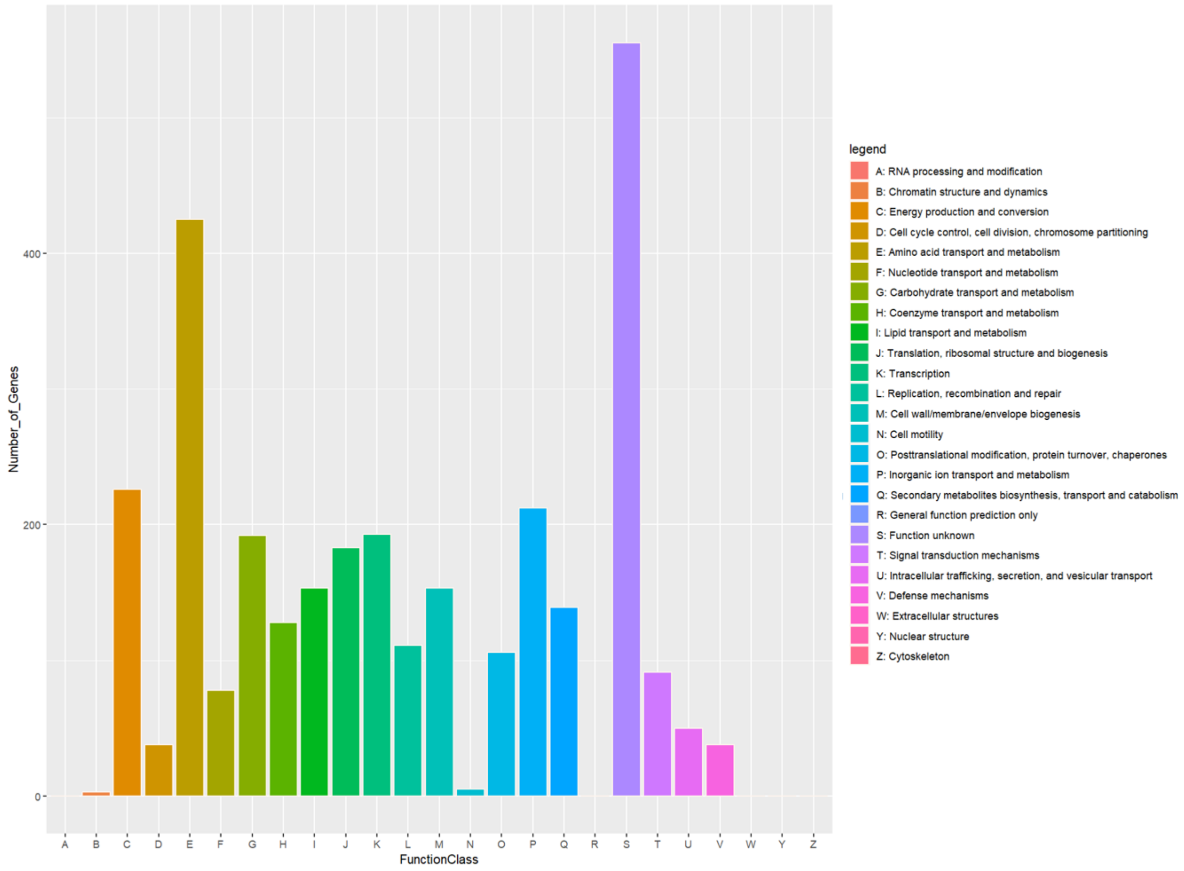

Fig S4. Venn diagram representing the orthologous gene clusters between the strain CAU 1616^T and reference strains. (except for *F. halophila*, for which the genomic data were absent).

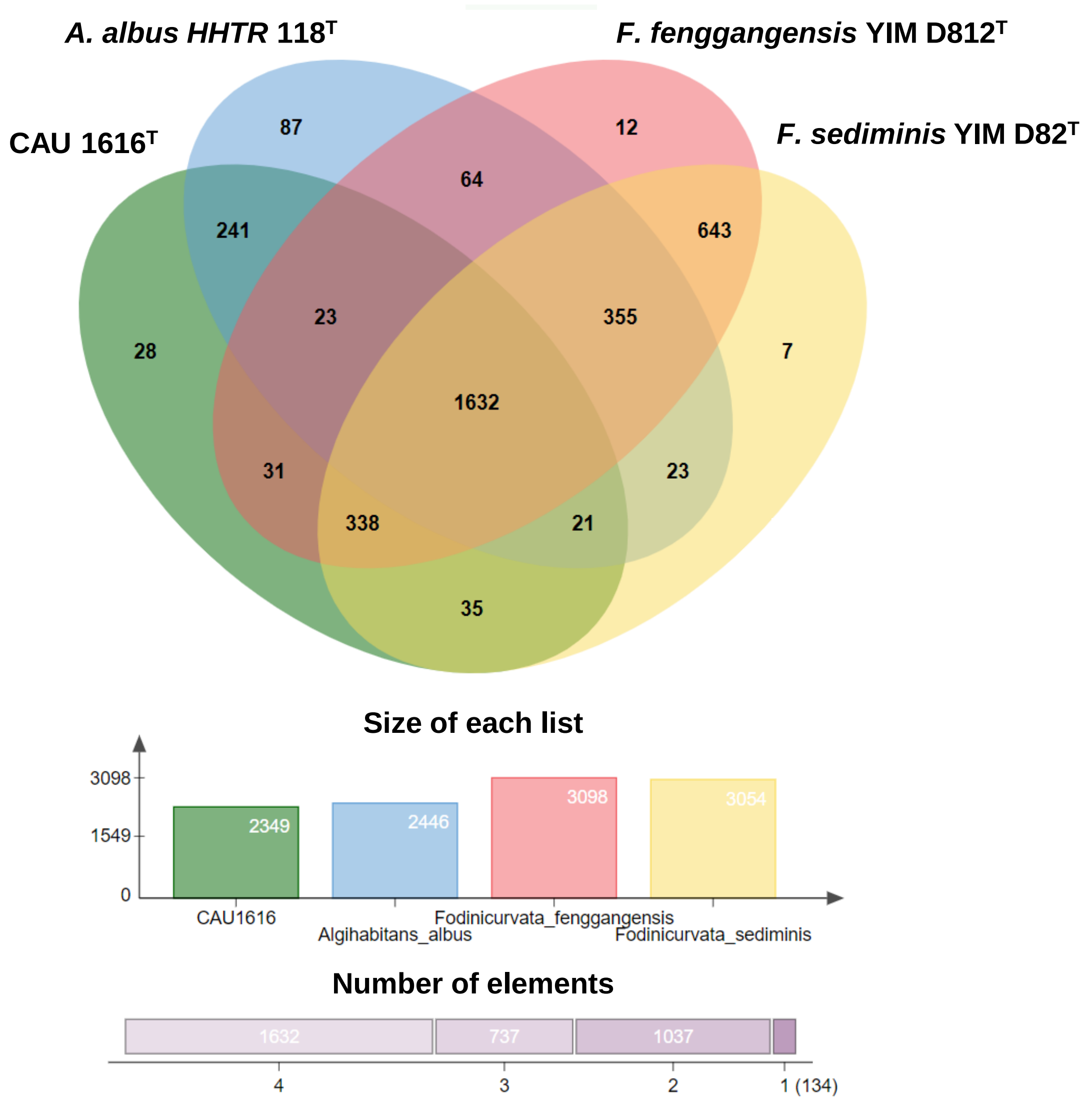

Fig S5. Transmission electron microscopy (TEM) image showed the characteristic morphology of negatively stained cells for CAU 1616^T. Bar, 1000 nm.

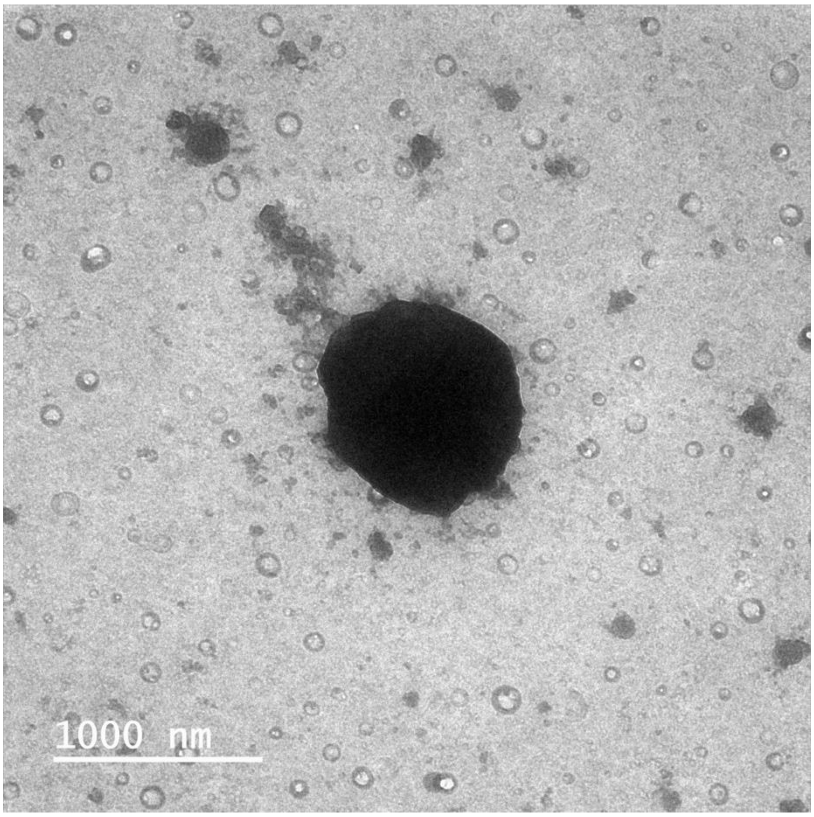

Table S1. Genomic features of strain CAU 1616^T with phylogenetically close strains (only genome available on NCBI). Strains: 1, CAU 1616^T; 2, *F. fenggangensis* DSM 21160^T; 3, *F. sediminis* DSM 21159^T; 4, *A. albus* HHTR 118^T.

**Genomic characteristics**	**1**	**2**	**3**	**4**
Accession number	GCF_029222965.1	GCF_000686045.1	GCF_000420625.1	GCF_003572205.1
Genomic size (Mbp)	3.5	3.8	3.7	4.7
DNA G+C contents (%)	65.1	61.0	60.6	64.9
Contig N50* (kbp)	385.8	211.2	551.5	741.1
Contig L50**	4	5	3	3
Number of CDSs***	3,218	3,569	3,475	4,351
Number of contigs	29	37	20	20
Number of rRNA	5	9	6	4
Number of tRNA	46	52	48	45

*N50, sequence length of the shortest contig at 50% of the total assembly length

**L50, count of smallest number of contigs whose length sum makes up half of genome size

***CDSs, coding sequences

Table S2. Distribution of COG categories in strain CAU 1616^T and closely related strains. Strains: 1, CAU 1616^T; 2, *F. fenggangensis* DSM 21160^T; 3, *F. sediminis* DSM 21159^T; 4, *A. albus* HHTR 118^T. (except for *F. halophila*, for which the genomic data were absent).

**COG Category**		**1**	**2**	**3**	**4**
POORLY CHARACTERIZED	(S) Function unknown	555	647	620	741
	(R) General function prediction only	0	0	0	0
INFORMATION STORAGE AND PROCESSING	(L) Replication, recombination and repair	111	143	135	120
	(K) Transcription	193	247	234	311
	(J) Translation, ribosomal structure and biogenesis	183	188	188	198
	(B) Chromatin structure and dynamics	3	4	4	4
	(A) RNA processing and modification	0	0	0	0
METABOLISM	(Q) Secondary metabolites biosynthesis, transport and catabolism	139	164	158	197
	(P) Inorganic ion transport and metabolism	212	195	169	233
	(I) Lipid transport and metabolism	153	141	139	218
	(H) Coenzyme transport and metabolism	128	146	150	179
	(G) Carbohydrate transport and metabolism	192	183	173	263
	(F) Nucleotide transport and metabolism	78	70	72	74
	(E) Amino acid transport and metabolism	425	480	468	492
	(C) Energy production and conversion	226	196	186	296
	(Z) Cytoskeleton	0	1	1	0

CELLULAR PROCESSES AND SIGNALING	(Y) Nuclear structure	0	0	0	0
	(W) Extracellular structures	0	0	0	0
	(V) Defense mechanisms	38	36	33	50
	(U) Intracellular trafficking, secretion, and vesicular transport	50	51	49	63
	(T) Signal transduction mechanisms	91	157	148	169
	(O) Posttranslational modification, protein turnover, chaperones	106	125	120	132
	(N) Cell motility	5	59	55	62
	(M) Cell wall/membrane/envelope biogenesis	153	152	153	208
	(D) Cell cycle control, cell division, chromosome partitioning	38	39	37	33

Table S3. RAST (Rapid Annotation Subsystem Technology) category distribution of strain CAU 1616^{T} and reference strains. Strains: 1, CAU 1616^{T}; 2, *F. fenggangensis* DSM 21160^{T}; 3, *F. sediminis* DSM 21159^{T}; 4, *A. albus* HHTR 118^{T}. (except for *F. halophila*, for which the genomic data were absent). The predominant subsystems (over 100 genes) are highlighted in bold.

**Subsystem feature counts**	**1**	**2**	**3**	**4**
Cofactors, vitamins, prosthetic groups, pigments	**100**	**142**	**122**	**188**
Cell wall and capsule	24	21	19	34
Virulence, disease and defence	31	35	31	35
Potassium metabolism	3	2	3	4
Photosynthesis	0	0	0	9
Miscellaneous	18	20	24	30
Phages, prophages, transposable elements, plasmids	2	4	2	2
Membrane transport	98	94	97	**146**
Iron acquisition and metabolism	6	4	6	7
RNA metabolism	37	39	38	36
Nucleosides and nucleotides	72	59	65	83
Protein metabolism	**184**	**182**	**180**	**176**
Cell division and cell cycle	0	0	0	0
Motility and chemotaxis	0	14	14	15

Regulation and cell signaling	12	23	22	27
Secondary metabolism	4	6	5	4
DNA metabolism	51	61	60	52
Fatty acids, lipids and isoprenoids	60	72	49	93
Nitrogen metabolism	23	23	28	17
Dormancy and sporulation	1	1	1	3
Respiration	86	94	95	97
Stress response	63	81	80	63
Metabolism of aromatic compounds	10	7	5	52
Amino acids and derivatives	**279**	**230**	**228**	**267**
Sulfur metabolism	8	6	5	12
Phosphorus metabolism	20	19	20	19
Carbohydrates	**164**	**153**	**142**	**198**

Table S4. Comparison of the potassium homeostasis protein in the strain CAU 1616^T and reference strains. Strains: 1, CAU 1616^T; 2, *F. fenggangensis* DSM 21160^T; 3, *F. sediminis* DSM 21159^T; 4, *A. albus* HHTR 118^T. (except for *F. halophila*, for which the genomic data were absent)

**Subsystem**	**Functional roles**	**Protein**	**1**	**2**	**3**	**4**
Potassium homeostasis	Large-conductance mechanosensitive channel	MSC	+	–	–	–
	KBP-type peptidyl-prolyl cis-trans isomerase	SlyD	+	–	–	–
	Potassium efflux system kefA protein	kefA	+	+	+	+

Table S5. Comparison of carbohydrate-active enzymes (CAZymes) between CAU 1616^T and closely related species. GH, Glycoside Hydrolases ; GT, GlycosylTransferase ; PL, Polysaccharide Lysases ; CE, Carbohydrate Esterases ; CBM, Carbohydrate-Binding Modules ; AA, Auxiliary Activities.

CAZymes	GH	GT	PL	CE	CBM	AA
***Aquibaculum arenosum* CAU 1616^T**	16	23	0	1	1	9
F. fenggangensis DSM 21160^T	11	20	0	3	0	8
F. sediminis DSM 21159^T	8	16	0	3	0	6
A. albus HHTR 118^T	14	35	0	4	0	10

Table S6. Number of predicted secondary metabolite biosynthetic gene clusters (smBGC) of CAU 1616^T genome. The BGCs were determined using antiSMASH 7.0

Cluster	SmBGC type	From	To	Most similar known cluster	Core biosynthetic gene	Additional biosynthetic gene
1	RRE-containing	76,721	97,926	Sunshinamide (25%)	1	4
2	T3PKS	170,962	212,014	-	1	5
3	NAGGN	26,823	41,557	-	3	1
4	NRPS-like	30,521	347,163	-	1	8
5	Terpene	49,978	70,862	-	1	4
6	T1PKS	1	30,049	Entolysin (13%)	1	5
